# Early Preinvasive Lesions in Ovarian Cancer

**DOI:** 10.1155/2014/639252

**Published:** 2014-04-08

**Authors:** Gautier Chene, Gery Lamblin, Karine Le Bail-Carval, Philippe Chabert, Naoual Bakrin, Georges Mellier

**Affiliations:** Department of Gynecology, Hôpital Femme Mère Enfant (HFME), University Hospital Lyon, 69000 Lyon, France

## Abstract

Faced with the catastrophic prognosis for ovarian cancer due to the fact that it is most often diagnosed late at the peritoneal carcinomatosis stage, screening and early detection could probably reduce the mortality rate. A better understanding of the molecular characteristics of the different ovarian cancer subtypes and their specific molecular signatures is indispensable prior to development of new screening strategies. We discuss here the early natural history of ovarian cancer and its origins.

## 1. Introduction

Epithelial ovarian cancer (90% of all ovarian cancers) is most often diagnosed at the advanced stage of peritoneal carcinomatosis with a poor prognosis (5-year survival rate 30 to 35%). Different epithelial subtypes have been defined: serous (50–70%), endometrioid (10–25%), clear cell (10%), and mucinous (5%). The serous subtypes can be subdivided into high-grade (75%) and low-grade serous epithelial ovarian cancer (25%) [1].

A dualistic model has been proposed, separating type 1 tumours (low-grade serous, mucinous, low-grade endometrioid, and clear cell tumours), with a relatively slow clinical evolution and weak response to platinum salts, from type 2 tumours (high-grade serous, high-grade endometrioid, nondifferentiated tumours, and carcinosarcoma) which develop rapidly, are frequently metastatic at the time of diagnosis, and are initially sensitive to platinum salts [2]. Type 2 tumours are characterised by p53 mutations in over 80% of cases, frequent alterations of BRCA expression (BRCA mutations in most hereditary predispositions and epigenetic inactivation in about 50% of sporadic carcinoma), and, unlike type 1 tumours, high-level chromosomal instability [3, 4]. However, the molecular profiles of type 1 tumours differ from each other (KRAS, BRAF, ERBB2, and PIK3CA mutations for low-grade serous carcinoma, ARID1A, CTNNB1, PTEN, PIK3CA, and PPP2R1A mutations for the endometrioid subtypes, ARID1A, PIK3CA, ZNF217, and PPP2R1A mutations for clear cell carcinoma, and KRAS and HER2 mutations for mucinous carcinoma). A more complex molecular model should be considered, including the 5 different histotypes and their various molecular signatures in order to better describe the heterogeneous nature of ovarian epithelial cancer along with the various specific targeted therapies [5, 6].

Many references in the literature allow a clearer distinction to be drawn between early lesions and high- and low-grade serous, endometrioid, and clear cell cancers. We will now present and discuss these points.

## 2. High-Grade Serous Ovarian Cancer

### 2.1. Ovarian Hypothesis: Compare Figure 1

In 1971, Fathalla [7] developed the theory of incessant ovulation after noting the high frequency of ovarian cancer in nulliparous women along with the protective role of oral contraception, pregnancy, and breastfeeding thanks to their inhibition of ovulation [8]; repeated ovulations could result in trauma to the ovarian epithelial surface. During healing, ovarian epithelium inclusion cysts could form and be affected by cellular and hormonal growth factors in the stromal microenvironment, resulting in a prelude to neoplastic transformation.

Indeed, at experimental level, the ovarian surface epithelium (OSE) has been found to play an important role: inhibition of ovulation by surgical ablation of surface epithelium in frog* in vitro *[11], and in sheep* in vivo* [12], along with proliferative ovarian epithelial activity located at the point of ovulatory trauma in rabbit [13]. Another argument in favour of this ovarian postulate is the high incidence of peritoneal carcinomatosis of ovarian origin in battery hens (repeated ovulation every 28 hours with a 30 to 40% spontaneous rate of peritoneal carcinosis at the age of 4 years) [14].

Surface epithelium presents, moreover, a mixed phenotype [15–17]: epithelial (types 7, 8, 18, 19 keratin, mucin, laminin, and type IV collagen) and mesenchymal (N-cadherine, types I and III collagen, and vimentin). Its plasticity has been shown in culture. Conversion to the mesenchymal phenotype slows with the progression of neoplasia such that eventually the epithelial phenotype alone remains, modified with the formation of glandular and papillary structures. This neoplastic epithelium then acquires the marker of epithelial differentiation, E-cadherin, while expression of C-met growth factor receptor is increased, which contributes to tumour growth [18].

Moreover, it seems that OSE expresses stem-cell markers (NANOG, SFRP1, JHX9, and ALDH1) that could confer the capacity to undergo neoplastic transformation [19, 20]. Auersperg explains that OSE may be a stem-cell niche [19]. The stem-cell profile of OSE that lines cortical inclusion cysts is different from the OSE on the ovarian surface. It may be a proof of preinvasive transformation [20].

This all underlines the essential interaction between OSE and the stromal microenvironment. Stromal hyperactivity affects the healing of the ovulation trauma due to epithelial proliferation stimulated by various cytokines and growth factors [21]. The dynamic interaction between OSE and underlying ovarian stroma thus appears to be the origin of epithelial differentiation, mullerian metaplasia [22, 23], and finally malignant transformation [24].

The concept of precancerous ovarian lesions called ovarian epithelial dysplasia was initially described in ovaries with a genetic risk (BRCA mutation) [25–30]. Given that these ovaries could change to high-grade serous cancer if prophylactic oophorectomy was not applied, epithelial dysplasia was logically considered to be a preinvasive lesion.

Similar dysplastic lesions were also revealed in areas adjacent to ovarian cancer and also in the contralateral ovary in case of unilateral ovarian cancer without any genetic predisposition [31–33].

In view of the difficult histopathological diagnosis, several measurement scales have been described.

- Salazar's score [27], based on the presence of at least three of the following criteria (sensitivity: 75%; specificity: 90%): inclusion cysts, epithelial pseudostratification, surface papillomatosis, deep invaginations, and stromal hyperplasia.
- Plaxe's score [31], based on the presence of the following four criteria (sensitivity: 98%; specificity: 100%): loss of polarity, epithelial stratification, cellular pleomorphism, and irregular chromatin distribution.

These two dysplasia assessment scales present the advantage of being simple but are incomplete (no nuclear criterion in the score proposed by Salazar et al. [27] and no criteria based on the physiopathology of dysplasia such as inclusion cysts, deep epithelial invaginations, and stromal dysplasia in the score proposed by Plaxe et al. [31]).

(iii) Deligdisch's score [34], based on the presence of deep surface epithelial invaginations, epithelial inclusion cysts, cortical stromal hyperplasia, psammoma bodies, surface papillomatosis, epithelial stratification, nuclear atypia, pleomorphism, stromal hyperplasia, and loss of polarity.
(iv) Our assessment scale [35, 36]; we have adapted the Deligdisch score by adding two severity levels for each of the abnormalities.

The metrological pertinence of this scale is proved by its reproducibility, the coherence of results when analysed by different pathologists, and its clinical-pathological validity.

Finally we used an ROC curve to establish the minimum threshold value showing a statistically significant difference with controls, thus making it possible to do without a control group; the cutoff level is 8 (sensitivity: 60%; specificity: 93.3%) [37].

Despite these assessment scales, histopathological diagnosis is still tricky. The two immunohistochemical markers p53 and Ki67 are described as being significantly expressed in ovarian cancer tissues compared with controls. The postulate that these markers would be expressed in dysplastic tissues could thus confirm their preinvasive nature and help in the diagnosis. Schlosshauer et al. [34] found an increasing gradient in these two markers, comparing normal ovarian epithelium (low expression), dysplastic epithelium (high expression), and ovarian cancer (even higher expression), which could also be a molecular argument in favour of the existence of dysplasia.

### 2.2. Tubal Hypothesis: Compare Figure 1

On the basis of the protective role of tubal ligature (a recent meta-analysis of tubal ligation and ovarian cancer found a 34% reduction in risk, essentially for endometrioid (RR = 0.4) and serous (RR = 0.73) cancers [38]) and of hysterectomy (preventing exposure to irritant genital tract germs) [39–41], the hypothesis of chronic pelvic inflammation was proposed. Indeed, there may be an association between upper-genital tract infections (in particular Chlamydia trachomatis and Mycoplasma genitalium) and epithelial ovarian tumours [42]. Taken together, the chronic inflammatory theory may be reinterpreted with the blame placed on the fallopian tube; the protective roles of tubal ligature or hysterectomy with salpingectomy could be explained by the absence of retrograde flow of inflammatory mediators. In opposition, the distal end of the fallopian tube could be exposed to inflammatory agents resulting in possibly repetitive damages and leading to precancerous lesions (see below).

New histopathological analyses with sampling of prophylactic adnexectomy tissues allowed up to 10% occult cancers to be detected, 57% to 100% of which were located in the fallopian tube fimbriae (serous tubal intraepithelial carcinoma or STIC) [43–49]. These lesions consist of nonciliated cells exhibiting 3 or more of the following features: abnormal chromatin pattern, nuclear enlargement, marked nuclear pleomorphism, epithelial stratification and/or loss of polarity, and nuclear moulding. They are also characterised by high immunohistochemical expression of TP53 (expression level between 80% and 92%) and highly positive levels for proliferation marker Ki67 and DNA double-strand break marker *γ*-H2AX [43–49].

Other even earlier tubal lesions have also been described. Serous tubal intraepithelial lesions or STIL [1] (also called TILT, tubal intraepithelial lesions in transition, by certain authors), tubal dysplasia, and tubal epithelial atypicalities [50], with not forgetting proliferative p53 signatures have been described but without any precise consensual definition (a morphological and molecular spectrum between p53 signatures and STICs).

Prior to STIL lesions, a succession of at least 12 secretory cells with intense nuclear p53 staining and a low proliferative index, named p53 signature, has been described. These lesions are located most often in the distal fallopian tube. It must be noted, however, that these p53 signatures can also be found in 50% of normal control tubes; it is not known currently which p53 signatures will progress towards STIL and then STIC and those that will remain benign [51].

**Figure 1:**
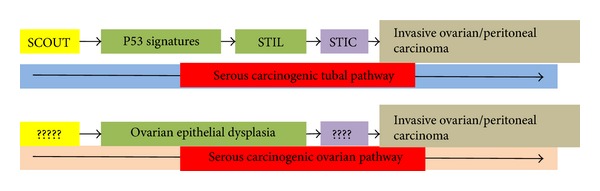
Comparison of the tubal pathway versus the ovarian pathway. The potential serous carcinogenic tubal sequence in comparison with the potential serous carcinogenic ovarian sequence. Note that SCOUT lesions (the earliest precursor lesion) could develop into other types of preinvasive lesions, p53 signature, and then STIL and STIC. The STIC would then easily metastasize in the ovary and adjacent peritoneum. By contrast, only the ovarian epithelial dysplasia is described as an ovarian preinvasive lesion. This figure raises the question of the interaction and molecular mechanisms between the fallopian tube and the ovary.

Finally, another extremely early abnormality, termed SCOUT (secretory cell outgrowths), was characterised by a succession of at least 30 pseudostratified secretory epithelial cells with a low expression of PAX2, PTEN, and Ki67 and with no p53 mutation. SCOUT can be located anywhere in the tube [52–56]. Although their meaning is not yet clear, certain authors believe that SCOUT are extremely early precursor lesions that could develop into other types of preinvasive lesions: p53 signature and then STIL and STIC. The STIC would then easily metastasize in the ovary and adjacent peritoneum [57] (compare Figure 1).

Several series of sporadic high-grade serous ovarian and serous peritoneal cancers (without BRCA mutation) were reanalysed and revealed the presence of the same serous carcinogenic sequence in almost 50% of cases, raising suspicions concerning the tubal origin of ovarian carcinogenesis [43, 58–62].

Mutation of TP53 tumour suppressor gene (at 17p13.1) is found in over 80% of cases of STICs. Even in sporadic cancers, the BRCA gene function is found to be changed in about 50% of cases (loss of heterozygosity and inactivation or hypermethylation of the promoter). How do p53 and BRCA interact? Is TP53 mutation required for activation of ovarian carcinogenesis? [63]

Functional organisation of the BRCA1 protein shows various domains (NSL involved in nuclear localisation, RING allowing interaction with other proteins, TAD with transcriptional activation functions). In the RING domain, a protein known as BARD1 (BRCA1-associated RING domain with similarities to BRCA1) plays an important antioncogenic role. BAP1 (BRCA1-associated protein 1) is another protein that interacts at this point and appears to be involved in suppressing cellular growth. TP53 can fix on the RING domain and interact with c-MYC, a transcription factor encouraging cellular proliferation. It can also fix on the TAD domain as C-terminal. BRCA1 thus appears to be a tumour suppression gene and a transcription regulator [63–65].

BRCA1, BRCA2 (via hRAD51), and TP53 interact in cooperation with the DNA repair channels (mainly by homologous recombination). DNA repair does not take place in mice with BRCA1 mutation; the result is the activation of TP53 and a halt in the cell cycle [66].

So inactivation of p53 in precancerous cells with BRCA mutations or functional abnormalities would avoid apoptosis and lead to cancer in association with other somatic mutations. Nevertheless, p53 immunohistochemical marking is negative in 15% of STICs [67]. This could be explained by the following.

- TP53 may occur upline from the protein segment targeted by the immunohistochemical analysis. Mutated and truncated proteins are not detectable by IHC.
- The location of the BRCA1 mutation is fundamental. If the BRCA1 mutation does not affect one of the p53 link domains, it can be supposed that other molecular abnormalities would be necessary to trigger the carcinogenic process. For example, 95% of tumours with BRCA1 mutation at 185delAG (the same level than a p53 domain) have a p53 mutation, while mutations upline appear to be less frequently associated with p53 abnormalities [68].
- p53 malfunction (without mutation) via abnormal synthesis of p53 binding protein or increased ubiquitination.
- Other genes that interact with TP53 may have mutated. Mutations of p21, ATM, CHK2, MDM2, and so forth (genes belonging to the DNA repair pathways) are enough to deactivate p53. Similarly various proteins in the cell cycle such as cyclin D1 or CDK4 may be altered and result in carcinogenesis [69, 70].

### 2.3. Tubal or Ovarian Origin?

We will now discuss the various arguments in favour of a tubal or ovarian origin for high-grade serous cancer.

#### 2.3.1. Embryological Argument

OSE and the fimbrial epithelium derived from the mullerian ducts have the same embryonic origin in the coelomic epithelium [20].

#### 2.3.2. Historical and Technical Arguments

*In Favour of a Tubal Origin*. Historically, ovarian dysplasia was described before the discovery of tubal lesions. The fallopian tubes were studied very little if not at all. So it cannot be excluded that STIC lesions may have been the origin of ovarian dysplasia lesions [71].

*In Favour of an Ovarian Origin*. Simple manipulation of the ovarian surface (resulting in microtrauma to the epithelium due to its extreme fragility) prevents ovulation in the pig [72]. It is often extremely difficult to achieve a complete histopathological analysis of OSE. So it cannot be excluded that dysplastic lesions may have been torn away prior to pathological examination.

#### 2.3.3. Chronological Argument

*In Favour of a Tubal Origin*. The presence of STICs without ovarian cancer occurs in specimens of prophylactic oophorectomy for genetic risk [47–49].

*In Favour of an Ovarian Origin*. The presence of ovarian dysplasia occurs with neither associated tubal lesions nor ovarian cancer [25, 35].

#### 2.3.4. Molecular Arguments

*In Favour of a Tubal Origin*. There were identical molecular profiles for STICs and synchronous high-grade serous ovarian or serous peritoneal cancers: identical p53 mutations in 93% of cases (missense and slice/frameshift mutations in 61% of cases) [73], overexpression of RSF-1, cyclin E, p16, FASN, stathmin, and laminin *γ* 1 genes, suggesting clonal expansion starting from tubal STIC lesions [74, 75].

*In Favour of an Ovarian Origin*. There is a similar immunohistochemical and molecular profile for preinvasive ovarian lesions and high-grade serous ovarian cancers: strong expression of p53, Ki67, Akt, and COX-2 and loss of Dab2 expression [34, 76, 77].

#### 2.3.5. Stem Cell Arguments

*In Favour of a Tubal Origin*. Stem cells may be distributed throughout the fallopian tube, but they may occur particularly in the distal parts (CD44, KRT5, integrin *α*6, NANOG, SFRP1, LHX9, and ALDH1 positive cells). This may explain why STICs are located in the fimbriae and this may suggest a triggering role in ovarian carcinogenesis [19, 78].

*In Favour of an Ovarian Origin*. OSE may be a stem-cell niche (high expression of NANOG, SFRP1, LHX9, and ALDH1), suggesting a triggering role in ovarian carcinogenesis [19].

#### 2.3.6. Genomic Instability: Compare Figure 2

*In Favour of a Tubal Origin*. Genomic instability is one of the main molecular characteristics of high-grade serous lesions and is present right from the STICs stage, as shown by the high expression of *γ*H2AX, genomic instabilities (visualized by comparative genomic hybridization (CGH)), and telomere shortening. We showed recently that STICs have the shortest telomeres compared with ovarian dysplasia lesions and invasive cancers. We proposed the hypothesis that STICs could correspond to the telomere crisis period just before genomic stabilization in ovarian invasive cancer due to telomerase stabilization. Moreover we found genomic imbalances that could be involved in transcriptional regulation, regulation of DNA damage response, and apoptosis [9, 79]. We found also an activation of the ATM-CHK2 signalling pathway in STIC lesions as it is the case in most of the earliest stages of human carcinogenesis [10].

*In Favour of an Ovarian Origin*. Genomic instability was found in ovarian dysplasia lesions in the form of shortening of telomeres and genomic alterations (visualized by array CGH) [9]. Telomeres were shorter than those in ovarian carcinoma but longer than those in STICs. Moreover, telomeres were significantly shorter in dysplastic epithelium with BRCA1 mutation than in those with BRCA2 mutation, probably because of the impact of BRCA1 in telomere maintenance mechanisms. We found also subtle genomic rearrangements that could be involved in the DNA damage response and in the p53 signalling pathway.

#### 2.3.7. Experimental Arguments

*In Favour of a Tubal Origin*. Animal models of tumorigenesis use tubal epithelial cells and result in high-grade mullerian cancers similar to high-grade human ovarian serous cancers from a molecular point of view [80, 81]; another interesting study has recently described a high-grade serous ovarian cancer model with knockout of Brca1 or 2, TP 53, and Pten. With this murine model, the authors managed to demonstrate that high-grade serous carcinomas may originate in the fallopian tubal secretory cells through the serous carcinogenic sequence (serous tubal intraepithelial carcinomas as a precursor lesion) [82].

*In Favour of an Ovarian Origin*. Animal experimental tumorigenesis occurs in which ovarian surface epithelium is the origin of tumours with the same molecular characteristics as high-grade serous cancers [83, 84].

Finally it seems that there are as many arguments in favour of a tubal as those in favour of an ovarian origin and the conclusion should probably show that high-grade serous cancer has a dual origin. A tubal origin would be preponderant in case of genetic mutation, unlike in sporadic cases, where the origin would more probably be ovarian (there are no STICs in up to 40% of the genetic series and in 50 to 70% of the sporadic series).

Nevertheless, including cases of ovarian origin, certain authors suggest the hypothesis of interaction with the tube, referring again to the incessant ovulation theory, tumours could develop from ovarian inclusion cysts (high cell proliferation index and aneuploidy in inclusion cysts in opposition with OSE) [85]. These cysts would be formed by invagination of tubal epithelium during ovulation, proof of which would be the presence of CD3 T cells, CD8 T lymphocytes, and CD68R macrophages (absent in cells from OSE). These inclusion cysts containing tubal cells would be subjected to the stromal microenvironment which would encourage malignant transformation [86]. However, the ovulatory defect is very thin and heals very quickly. So it is difficult to imagine that tubal cells could penetrate to this level [87].

For type 1 tumours, the fallopian tube would also play an important role in the genesis of low-grade serous, endometrioid, and clear cells cancers.

## 3. Low-Grade Serous Cancer

There appears to be a morphological and molecular continuum marked by the the following sequence: serous cystadenoma/adenofibroma, then evolution towards typical serous borderline tumours and noninvasive micropapillary serous carcinoma, and finally invasive low-grade serous carcinoma. The origin of serous cystadenoma is still the subject of debate. Previously, it has been found that serous epithelial tumors (from cystadenomas to low-grade carcinoma) were significantly associated with a lesion called salpingolith (defined as luminal and mucosal calcifications surrounded by a mantle of bland tubal epithelium) [88]. Recently, the same authors designated the possibly precursor lesion more accurately and called it the papillary tubal hyperplasia (PTH). PTH is characterized by small rounded clusters of tubal epithelial cells and small papillae, with or without psammoma bodies, floating within the tubal lumen [88, 89]. One preliminary study alone reports a rate of PTH in 91% of cases (20/22) [89].

The carcinogenesis model would start with chronic inflammation leading to tubal hyperplasia, then papillary tubal hyperplasia (PTH), and finally extension to the ovary and adjacent peritoneum [89, 90]. For other authors, serous cystadenoma would arise from certain ovarian inclusion cysts with the following molecular profile: expression of PAX8 and tubulin and lack of expression of calretinin, for example, a tubal phenotype [91]. These inclusion cysts would have either a tubal origin, by endosalpingiosis (maybe tubal stem cells), or an ovarian origin, by invagination of OSE with subsequent tubal metaplasia. Because of the diminution of tubal ciliated cells, low-grade serous cancer may be the consequence of a clonal expansion of tubal secretory cells. Moreover it has been described as an association between SCOUT lesions (the same earlier precursors as it is the case in the high-grade serous carcinogenic sequence) and serous borderline tumours [92]. We could hypothesize that there could be an identical tubal origin with an orientation towards low-grade carcinoma (because of KRAS and BRAF mutations) or towards high-grade carcinoma (with TP53 mutations and genomic instability). Other studies are needed to define the accurate model for the origin and development of low-grade serous carcinoma.

## 4. Endometrioid and Clear Cell Cancers

Several cohort studies found a significant association between endometriosis and ovarian cancer (especially clear cell and endometrioid ovarian cancers) [93, 94]. Standardized incidence ratios (SIRs) with 95% confidence intervals were 1.9 (1.3–3.8) for Brinton et al. [93] and 1.3 (1.0–1.8) for Borgfeldt and Andolf [94]. The ovarian cancer risk seems to be higher among women with a long history of endometriosis [93].

Recently mutation of ARID1A gene (AT rich interactive domain 1A) was found in 41 to 57% of clear cell cancers and 30 to 48% of endometrioid cancers [95–97]. ARID1A is a tumour suppressor gene and encodes BAF 250a protein that is involved in the multiprotein SWI/SNF chromatin-remodelling complex. The SWI/SNF complex is involved in DNA repair, DNA synthesis, and genomic stability since it has been shown to target the chromatin at DNA double-strand break sites. ARID1A mutation may lead to cellular dysfunction as aberrant chromatin remodelling and alterations in the cell cycle checkpoints [98]. Moreover, loss of expression of this gene was recently found in benign endometriosis (20%) and endometriosis with atypicalities (38.5%) adjacent to malignant lesions (57.7%), suggesting that these cancers do not originate in the ovary but in the endometriosis itself [99]. Samartzis et al. [100] found loss of ARID1A/BAF 250a expression in presumably benign ovarian endometriomas (*n* = 3/20, 15%), particularly in the form of cell clusters that could suggest a clonal loss of BAF 250a and a risk of carcinogenic transformation.

Loss of ARD1A expression is therefore an early event in ovarian clear cell carcinoma and is often associated with PIK3CA mutations (*P* = 0.013) that could initiate tumour development [101].

A transcription factor, HNF-1*β* (hepatocyte nuclear factor-1*β*), is also overexpressed in clear cell carcinoma (92.3%), in atypical endometriosis (53.8%), and in benign distant endometriosis (33%) [102]. It has been proposed that clear cell carcinoma arises from the HNF-1*β* positive epithelial cells of endometriosis, whereas endometrioid carcinoma may arise from HNF-1*β* negative endometriosis.

The tube would play the role of conduit and could thus be involved in relation to retrograde menstrual flow which is one of the main hypotheses for the genesis of endometriosis [103].

## 5. Clinical Implications

Until the advent of new targeted therapies, the only way to improve the prognosis for ovarian cancer is early detection of these lesions [104].

We are eagerly awaiting the final results (scheduled for next year) of Ian Jacobs' series comparing the individual CA125 profile obtained by longitudinal follow-up of asymptomatic patients with CA125 profiles corresponding to indexed cases of ovarian cancer [105, 106]. If this algorithm is validated, it would provide the means for efficient screening.

A prospective study addressed the relationship between CA125 serum levels and dysplasia [107]; the absolute value of CA125 in dysplasia cases was predictive for ovarian cancer (*P* = .003). The sensitivity, specificity, and positive predictive value of a value of at least 14 U/mL in the group of patients with a BRCA mutation were, respectively, 54%, 75%, and 47%. Despite the fact that the authors suggest that CA125 could help in the decision for prophylactic adnexectomy, the 3 occult cancers in this series presented a normal level (15 U/mL, 11–20).

The conventional Papanicolaou smear probably deserves to be developed not only for early detection of ovarian serous borderline tumours [108] but also for invasive cancers; 41% (9/22) of ovarian cancers were identified using a panel of mutated genes from liquid Papanicolaou smear specimens [109].

Concerning invasive methods [110], peritoneal cytology should probably be mandatory in any patient with a risk of ovarian cancer (BRCA mutation) and undergoing prophylactic adnexectomy; in a recent review of the literature, 10 (32.2%) out of 31 cases of STICs discovered in a context of prophylactic adnexectomy showed positive cytology. It is probable that this rate could be improved by lavage of the adnexa using normal saline [111]. Moreover a proteomic study is under way at present, based on lavage of the fimbriae during laparoscopy.

Optical biopsy is an imaging technique that allows for real-time 3D display of tissue sections [112]. It provides excellent results for detection of preinvasive gastrointestinal lesions [113]. In gynaecology [112], optical biopsy appears to improve the performance of colposcopy for detection of cervical intraepithelial neoplasia.

McAlpine et al. [114] were able to view STIC tubal lesions with 73% sensitivity, 83% specificity, 57% positive predictive value, and 91% negative predictive value.

Now that probes have been miniaturized and noncytotoxic contrast media are available; confocal microlaparoscopy (optical biopsy combined with laparoscopy) could certainly be useful in patients with a genetic risk of ovarian cancer (BRCA mutations) and who want to remain fertile, by allowing a precise histopathological diagnosis for the ovaries and tubes in real time and* in vivo.*

Finally, because of the role of the fallopian tube in ovarian carcinogenesis, certain authors have suggested exclusive salpingectomy without associated oophorectomy [115].

Although conservation of the ovaries prior to the menopause avoids the cardiovascular, osteoporotic, and psychosexual morbidity and mortality induced by surgical castration, a carcinological risk remains, such as ovarian cancer of ovarian origin and also breast cancer (the breast cancer risk drops by 35 to 50% in patients undergoing bilateral oophorectomy prior to age 50, which is all the more important when there is a BRCA mutation [116, 117]).

Kwon et al. [118] have developed a simulation model comparing three strategies in the BRCA population: bilateral salpingo-oophorectomy, bilateral salpingectomy, and bilateral salpingectomy with delayed oophorectomy. The authors conclude that prophylactic adnexectomy is the best in terms of reducing the risk of ovarian and breast cancer. However, bilateral salpingectomy with delayed oophorectomy could be an interesting option in terms of cost-effective strategy and higher quality of life.

## 6. Conclusions

**Figure 3:**
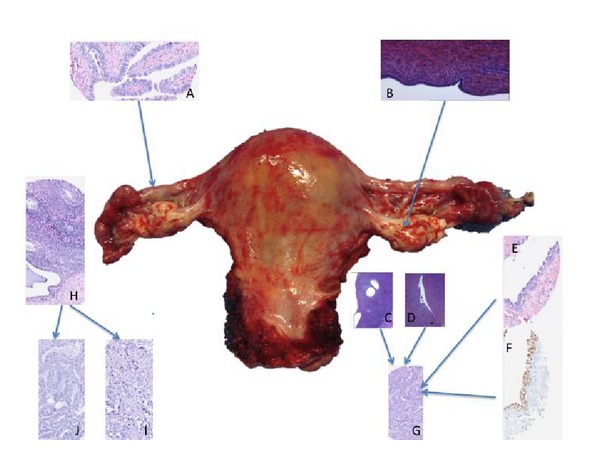
Precursor and invasive lesions in high-grade serous, clear cell, and endometrioid carcinoma. Note. Normal fallopian tube (A) and ovarian surface epithelium (B). High-grade serous ovarian carcinoma (G) may arise from ovarian dysplasia (inclusion cysts in C, deep cortical invaginations in D) and/or from tubal preinvasive lesions (STIC, H&E in E, and high immunohistochemical expression of P53 in F). On the other side, endometriosis (H) may develop in endometrioid carcinoma (J) or clear cell carcinoma (I). With this picture, we would like to show the thin anatomical connection between ovary and fallopian tube and their implications in ovarian carcinogenesis.

There are conflicting data concerning the tubal or ovarian origin of ovarian cancer. (compare Figure 3). It is now clear that there is not just one type of ovarian cancer, but that it is a heterogeneous disease corresponding to distinct molecular signatures. In terms of statistical power, precursor lesions are in fact rare. Further studies are still needed to better understand the various preneoplastic phases of ovarian cancer and the interaction between the fallopian tube and the ovary.

## Figures and Tables

**Figure 2:**
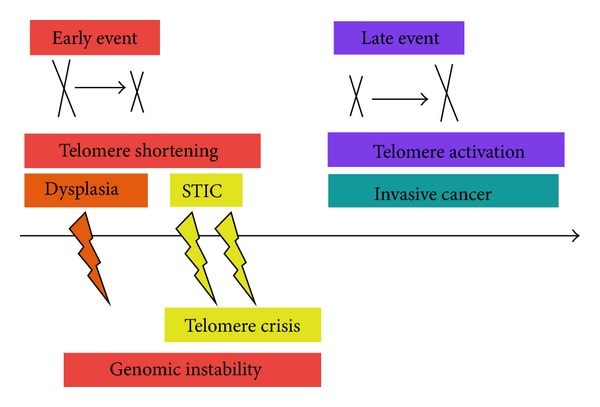
Schematic representation of telomere length and genomic instability steps ovarian/tubal carcinogenesis. Telomere shortening and genetic instability are one of the first events in ovarian carcinogenesis. The STIC lesions have the shortest telomeres followed by ovarian dysplasia. We have made the hypothesis that STIC lesions could be in a telomere crisis phase preceding genomic stabilization due to telomerase activation during the invasive stage. Therefore, telomerase activation could likely counterbalance the telomere shortening in order to represent an advantage in terms of tumor proliferation and escaping apoptosis [9, 10].
